# Life changes, self-prevention, knowledge and mental health among inflammatory bowel disease patients during COVID-19 pandemic: a cross-sectional study

**DOI:** 10.3389/fpubh.2024.1416880

**Published:** 2024-06-12

**Authors:** Shiwen He, Tao Xiao, Yingchun Xia

**Affiliations:** Nursing Department, The Third Xiangya Hospital, Central South University, Changsha, China

**Keywords:** COVID-19, anxiety and depression, life changes, self-prevention, inflammatory bowel disease

## Abstract

**Background:**

With the COVID-19 pandemic going to be COVID-19 endemic, the negative impact of COVID-19 on the mental health of IBD patients cannot be ignored. This study aimed to investigate the occurrence of anxiety and depression in IBD patients during the COVID-19 pandemic and analyze the factors associated with mental health.

**Methods:**

Patients registered at the IBD center were enrolled. Electronic questionnaires about the IBD patient’s demographic information, basic knowledge of COVID-19, public self-prevention measures, daily life changes, and anxiety and depression were distributed.

**Results:**

Two hundred and fifteen IBD patients finished this study and reported to have anxiety (27%) or depression (34%). During the COVID-19 pandemic, 10.2% of IBD patients reported their diet changes, 58.5% of IBD patients changed their daily physical activities from 3.27 ± 3.252 h to 2.30 ± 2.78 h, 33.7% of IBD patients changed their sleeping duration from 7.99 ± 1.322 h to 8.18 ± 1.447 h. IBD patients’ waiting time for admission (OR: 3.688, 95%CI: 1.003–13.554), regularly oral medicine administration (OR: 18.407, 95%CI: 1.975–171.530) and diet changes (OR: 6.167, 95%CI: 2.158–17.626) were positively correlated with anxiety or depression. IBD patients’ timely periodic infusion of biological agents (OR: 0.586, 95%CI: 0.413–0.830) was negatively correlated with anxiety or depression. IBD patients’ knowledge of COVID-19, public self-prevention, physical activities, and sleep duration changes showed no significant correlation with anxiety and depression, all *p* values > 0.05.

**Conclusion:**

The main factors of IBD patients’ mental health were diet changes, waiting time for admission, taking oral medicine regularly, and timely periodic infusions of biological agents. Ensuring the supply of routine treatment and medication for IBD patients and establishing systemic online IBD self-management programs would be the focus of major public health events.

## Introduction

World Health Organization (WHO) declared the Corona Virus Disease 2019 (COVID-19) pandemic on 11 March 2020 and declared COVID-19 over as a global health emergency on 5 May 2023. Because of high levels of reinfection in the Omicron, reinfection events would dominate future COVID-19 dynamics (1). Susceptible populations still need to focus on preventing Omicron, such as SARS-CoV-2.

Compared with the general population, the pooled incidence of COVID-19 in inflammatory bowel disease (IBD) patients was 18% higher than that of the general population (2). IBD consists of ulcerative colitis (UC) and Crohn’s disease (CD), which affect individuals from the entire age spectrum and have a substantial negative impact on quality of life (3). IBD has become a global disease with an accelerating incidence in newly industrialized countries in Africa, Asia, and South America (4). Immunosuppressant therapy, one of the main treatments for IBD patients, could significantly reduce the risk of hospital admission and surgery (5). IBD patients showed no significantly increased risk or aggravated outcomes during the COVID-19 pandemic (6). However, IBD patients treated with thiopurines, methotrexate, anti-tumor necrosis factor α agents, and corticosteroids had a higher incidence of SARS-CoV-2 infection after vaccination compared with healthy control subjects (7). Thus, stopping immunosuppressive treatments for all patients with IBD is challenging (5).

Before the COVID-19 pandemic, IBD patients had a prevalence rate of 32.1% for anxiety and 25.2% for depression, which were higher than those in the general population (8). For active IBD patients, the prevalence rate of anxiety and depression was up to 58 and 50% (9). During the COVID-19 pandemic, IBD patients had moderate to severe depression, anxiety, and stress with prevalence rates of 34.9, 32.0, and 29.7%, respectively (10). And 60.5% of IBD patients had at least moderate depression, anxiety, or stress (10). With the COVID-19 pandemic going to be COVID-19 endemic, the negative impact of COVID-19 on the mental health of IBD patients cannot be ignored.

Life changes during the COVID-19 pandemic were related to poor mental health in university students, such as giving up a daily routine, neglecting meals, tidiness, and hygiene (11). More than 50% of IBD patients did not change their IBD medication and interrupt IBD services, but they still reported a negative impact of COVID-19 on their quality of life (12). The health-related quality of life pre-COVID-19 decreased during the COVID-19 outbreak, and a lower health-related quality of life was associated with perceived stress in IBD patients (13). Social dysfunction caused by the lockdown was significantly related to COVID-19 anxiety (14). “staying at home,” “canceled events” and “increased workload” were the top three life changes during the COVID-19 pandemic for IBD patients with psychological distress (15).

Even though the above studies had reported some life changes and psychological stress experienced by IBD patients, the correlation between life changes, such as diet, physical activities, sleep changes, and mental health in IBD patients were less reported. Moreover, high levels of risk perceptions of contracting COVID-19 may increase an individual’s pandemic-related stress and contribute to negative mental health consequences (16). Thus, this study aimed to assess the knowledge of COVID-19, public self-prevention measures, daily life changes, and mental health in IBD patients.

## Materials and methods

### Participants and procedure

We conducted a cross-sectional survey at the Third Xiangya Hospital of Central South University, one of the midland diagnosis and treatment centers for IBD in China, between August 18, 2022, and October 18, 2022. All IBD patients registered in this center were recruited if they had no history of mental illness, were literate enough to read and write, and had and used a smartphone. IBD patients were excluded from this study if they: (1) had communication disorder or cognitive disorders, (2) had serious physical illness unable to complete the investigation, or (3) did not provide a consent form.

During the study period, the Chinese government published the ninth version of the COVID-19 prevention and control plan, which divided risk areas into high, medium, and low-risk based on the results of dynamic epidemiological investigation. People in areas with high or medium risk were encouraged to stay at home. People in low-risk areas must show the negative results of COVID-19 nucleic acid tests within 48 h when entering shopping malls, schools, and hospitals. Once the COVID-19 nucleic acid test was positive, the China Disease Prevention and Control Information System would report the activity trajectory within 2 h. The infected people would go to designated medical institutions or shelter hospitals for treatment (17).

This study was carried out through an online investigation. The study members explained the content and purpose of this study through phone calls or e-mails and invited IBD patients to finish the anonymous questionnaires. When IBD patients or guardians (patients <18 years old) agreed, study members sent the electronic questionnaires affiliated with the consent link to the patient’s mobile phone within 10 min, and the link was valid for 60 days. To reduce the dropout rate, we invited IBD patients to participate in this study when admitted during the investigation. We obtained ethics approval from the Third Xiangya Hospital of Central South University Ethics Board (快Ι22175).

### Sample size estimation

According to previous data (10), it is found that 60.5% of IBD patients had at least one mental health problem during COVID-19 pandemic. We required that with 95% confidence, the results need to fall within 15% of the overall truth rate. According to the calculation formula *N* = *Z*^2^_1-α/2_(1−*p*)/ε^2^γ, it is calculated that *N* = 112. Considering the 10% of attrition rate and the design efficiency, the questionnaires required for this cross-sectional study were at least 135 in total.

### Instruments

Two questionnaires were used in this study, one is a self-made questionnaire consisting of demographic information, life changes (nine questions), knowledge of COVID-19 (15 questions), and public self-protection measures (three questions), another is Hospital Anxiety and Depression Scale.

### Demographic information

Demographic information such as age, weight, gender, time of diagnosis, comorbidity, living area, educational background, marital status, monthly income, sources of getting COVID-19 knowledge, oral medicine administration, infusions of biological agents, and waiting time for admission were collected.

### Life changes

Participants reported if they had changed the frequency of food consumption, physical activity duration, sleeping duration, and medical treatment activities. Food consumption included the food categories in The Chinese Food Frequency Questionnaire: staple food (including rice, wheat flour, and other cereals), fresh vegetables, fresh fruit, meat (including pork, poultry, and fish), eggs/milk, and others (18). The definition of physical activities includes vigorous physical activities (such as heaving lifting, digging, aerobics, or fast bicycling), moderate physical activities (such as carrying light loads, bicycling at a regular pace, or doubles tennis), and walking (including walking at work, walking at home, walking to travel from place to place, and other walking) (19).

Participants reported food consumption changes by filling out the single-choice questions. “Whether your diet changed or not after the COVID-19 pandemic outbreak? (yes/no),” “If your diet changed, what kind of change in consumption of staple food, fresh vegetables/fruit, meat/eggs/milk, and others? (increased/ decreased).”

Sleep and physical activity changes were assessed by questions “How many hours of sleep did you get every night before the COVID-19 pandemic?,” “How many hours of sleep do you get every night during the COVID-19 pandemic?,” “How many hours did you spend on daily physical activities before the COVID-19 pandemic?,” and “How many hours do you spend on daily physical activities during the COVID-19 pandemic?”

Participants reported their medical treatment activities by answering the questions. Do you take your medication regularly during the COVID-19 pandemic (yes/no)? Do you get the infusion of biological agents on time during the COVID-19 pandemic (yes/no/ no need)? How long do you wait for admission (day)?

### Knowledge of COVID-19 and public self-protection measures

Self-made questionnaires were used to evaluate the patients’ knowledge of COVID-19 and their daily self-protection measures. The questionnaire about COVID-19 knowledge included 15 single-choice questions, 5 points for each question, with a total score of 75 points. The question” What are your major sources of COVID-19 knowledge?” was provided for participants. The questionnaire about public self-prevention measures consisted of three questions: “How many times a day have you monitored your temperature in the last 3 months?”; “How many times a week have you been out in the last 3 months?”; and “How many times a month have you been to the hospital in the last 3 months?”

### Hospital anxiety and depression scale

The Hospital Anxiety and Depression Scale was used to measure symptoms of anxiety and depression and consists of two scales and 14 items, with seven items for the anxiety scale (HADS-A) and seven items for the depression scale (HADS-D). Each item score ranges from 0 to 3, and each scale (HADS-A or HADS-D) values between 0 and 21. Three ranges for both of the scales: 0–7 (non-cases), 8–10 (doubtful cases), and 11–21 (cases). When a patient has scores ≥ 8, it indicates that the patient has symptoms of depression and anxiety, and intervention treatment is needed as soon as possible (20). The Chinese version of the HADS showed good reliability and sensitivity for correctly identifying psychiatric mood disorders (21).

### Statistical analysis

Data were analyzed by using SPSS 19.0 software. Frequencies and rates were used to describe the prevalence of anxiety or depression, patients’ demographic information, sources of getting COVID-19 knowledge, cognition of each COVID-19 topic, public self-prevention, and life changes. Mean ± SD was used to describe the scores in COVID-19 knowledge, HADS-A, and HADS-D. Mann–Whitney U was used to analyze the HADS scores among participants with different genders, chronic diseases, living areas, marital status, diets, physical activities, sleep duration, and oral medicine regularly, and compare the knowledge of COVID-19 between participants who had anxiety or depression. Kruskall-Wallis H was used to compare the HADS-A and HADS-D scores among participants with different diagnosis times, educational backgrounds, monthly income, public self-prevention, periodic infusion of biological agents, and waiting time for admission. The impact of demographic backgrounds, COVID-19 knowledge, public health prevention, and life changes on anxiety or depression were estimated using binary logistic regression. A value of *p* < 0.05 was considered statistically significant.

## Results

### Participant characteristics

**Figure 1:**
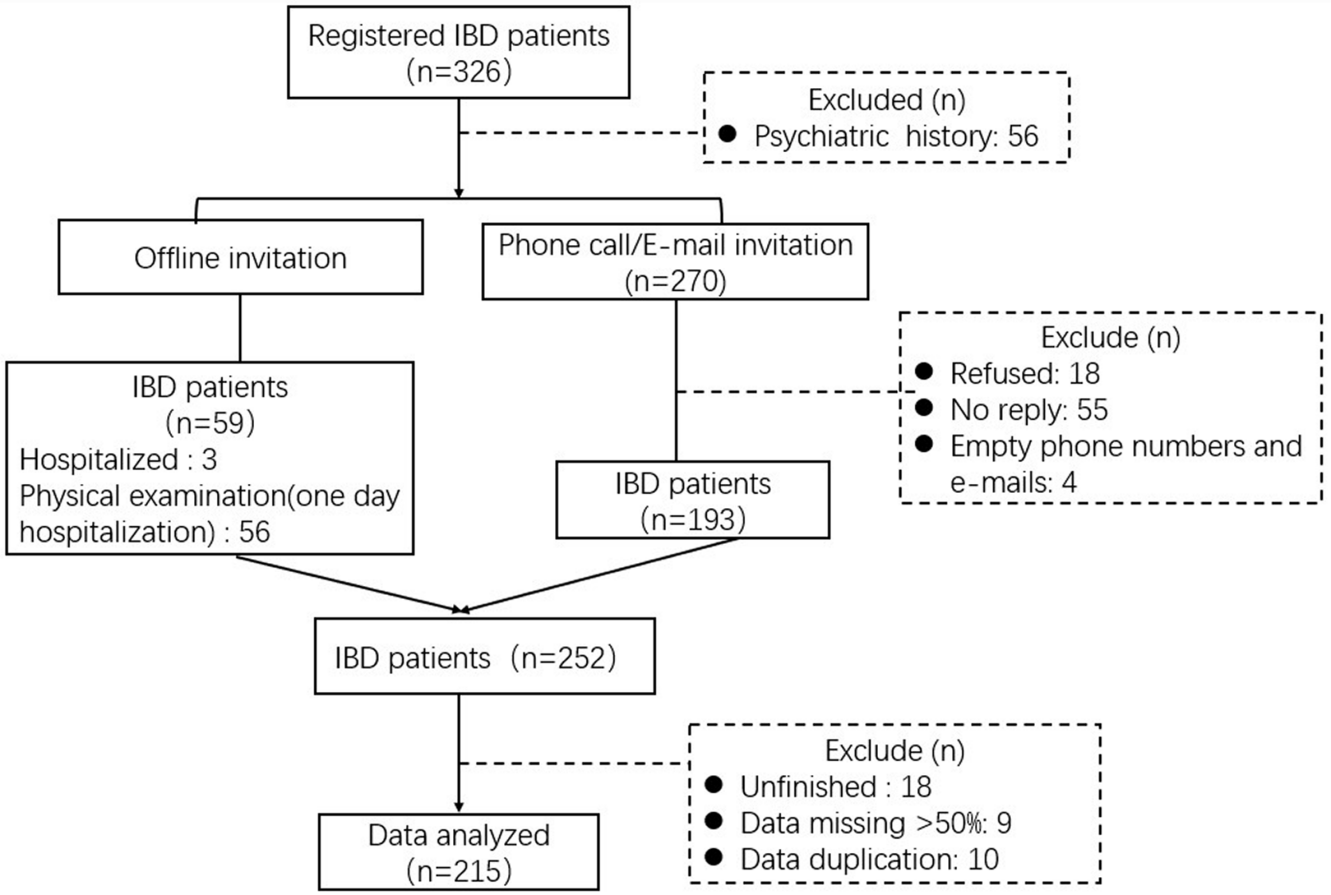
The progress of participants recruited.

During August and October 2022, we enrolled 270 patients with IBD. No patients were infected with COVID-19. Fifty-six IBD patients were diagnosed with mental illness before this investigation, and 18 IBD patients refused participation. Finally, 252 IBD patients participated in this study, and 215 questionnaires were analyzed after excluding 37 invalid questionnaires. The questionnaire response rate was 79.6%, detailed in Figure 1.

The IBD patients were 32.20 ± 11.96 years old and weighed 56.02 ± 9.74 kilograms. More than 50% of them were male, diagnosed with IBD more than 1 year, with no chronic disease, living in urban areas, married, and with less than 5,000 monthly incoming, detailed in Table 1.

**Table 1:** The demographic information and scores of HADS in IBD patients.

Characters		*n*	%	HADS-A		*p* values	HADS-D		*p* values
Gender	Male	158	73.5	5.46 ± 2.98	*Z* = −0.022	*p* = 0.982	6.02 ± 3.52	Z = −0.675	*p* = 0.5
	Female	57	26.5	5.30 ± 2.97			5.61 ± 3.17		
Diagnosis time	<1 year	75	34.9	5.23 ± 3.19	*H* = 0.613	*p* = 0.736	5.97 ± 3.67	*H* = 1.227	*p* = 0.542
	1–3 years	65	30.2	5.54 ± 3.18			6.17 ± 3.57		
	>3 years	75	34.9	5.51 ± 2.57			5.63 ± 3.06		
Complications of other diseases	Yes	38	17.7	6.11 ± 2.59	*Z* = −1.169	*p* = 0.243	6.68 ± 2.83	*Z* = −1.443	*p* = 0.149
	No	177	82.3	5.27 ± 3.04			5.75 ± 3.53		
Living areas	Urban	135	62.8	4.73 ± 3.14	*Z* = −3.952	**p < 0.001**	5.21 ± 3.38	*Z* = −4.008	**p < 0.001**
	Rural	80	37.2	6.58 ± 2.32			7.10 ± 3.20		
Education background	<High school	53	24.7	6.04 ± 2.93	*H* = 7.467	**p = 0.024**	7.40 ± 2.81	*H* = 18.718	**p < 0.001**
	High school	34	15.8	4.47 ± 2.59			4.29 ± 3.26		
	>High school	128	59.5	5.41 ± 3.04			5.73 ± 3.49		
Marital status	Unmarried	86	44.7	5.31 ± 3.07	*Z* = −0.012	*p* = 0.990	5.80 ± 3.58	*Z* = −0.183	*p* = 0.855
	Married	119	55.3	5.46 ± 2.85			5.88 ± 3.39		
Monthly income (RMB)	>5,000	41	19.1	3.98 ± 3.12	*H* = 14.106	**p = 0.001**	4.10 ± 3.41	*H* = 15.079	**p = 0.001**
	3,000–5,000	100	46.5	6.13 ± 2.71			6.68 ± 3.02		
	<3,000	74	34.4	5.26 ± 2.94			5.88 ± 3.60		

IBD, Inflammatory Bowel Disease. Significant of bold *p* values.

HADS-A, The Hospital Anxiety and Depression Scale-Anxiety.

HADS-D, The Hospital Anxiety and Depression Scale-Depression.

### The prevalence of anxiety and depression

Among the IBD patients, 27% of participants had depression (HADS-A scores ≥ 8), and 34% of participants had anxiety (HADS-D scores ≥ 8). As reported in Table 1, IBD patients who lived in rural areas, had an education background below high school and had a monthly income of less than 5,000 RMB showed higher scores for anxiety and depression.

### Life changes and correlation with mental health

10.2% of IBD patients reported their diet changes: staple food consumption increasing (*n* = 6, 2.8%), staple food consumption decreasing (*n* = 6, 2.8%), fresh vegetables and fruit consumption increasing (*n* = 10, 4.6%), fresh vegetables and fruit consumption decreasing (*n* = 2, 0.9%), meat/eggs/milk consumption increasing (*n* = 4, 1.8%), meat/eggs/milk consumption decreasing (*n* = 8, 3.7%), and others (*n* = 6, 2.8%).

58.5% of IBD patients changed their daily physical activities from 3.27 ± 3.252 h to 2.30 ± 2.78 h. And 33.7% of IBD patients changed their sleeping duration from 7.99 ± 1.322 h to 8.18 ± 1.447 h. 20.5 and 19.4% of IBD patients could not take oral medicine regularly or receive biological agents’ infusion on time. Their life changes and correlation with mental health were shown in Table 2.

**Table 2:** The correlations between life changes and mental health.

Life changes		*n*	%	HADS-A		*p* values	HADS-D		*p* values
Diet	Yes	22	10.2	7.27 ± 1.91	*Z* = −3.182	**p = 0.001**	7.09 ± 4.13	*Z* = −1.942	*p* = 0.136
	No	193	89.8	5.21 ± 3.00			5.78 ± 3.33		
Physical activities	Yes	121	58.5	5.62 ± 2.91	*Z* = −1.002	*p* = 0.316	6.24 ± 3.26	*Z* = −1.330	*p* = 0.184
	No	86	41.5	5.14 ± 3.15			5.49 ± 3.60		
Sleep duration	Yes	67	33.7	5.91 ± 2.95	*Z* = −1.967	**p = 0.049**	6.10 ± 3.92	*Z* = −0.304	*p* = 0.761
	No	132	66.3	5.08 ± 3.08			5.95 ± 3.22		
Medicine regularly	Yes	171	79.5	5.30 ± 3.01	*Z* = −1.069	*p* = 0.285	5.95 ± 3.45	*Z* = −0.473	*p* = 0.636
	No	44	20.5	5.89 ± 2.80			5.77 ± 3.38		
Periodic infusion of biological agents	On time	72	39.1	4.33 ± 3.51	*H* = 10.232	**p = 0.006**	4.67 ± 3.37	*H* = 21.307	**p < 0.001**
	Not on time	42	22.8	6.19 ± 2.96			7.86 ± 3.51		
	No need	70	38.1	5.60 ± 2.54			5.86 ± 3.21		
Waiting time for admission (day)	≤3 days	149	81.0	4.98 ± 3.07	*H* = 5.708	*p* = 0.058	5.50 ± 3.44	*H* = 7.879	**p = 0.019**
	4–6 days	21	11.4	6.19 ± 3.28			7.57 ± 3.89		
	≥7 days	14	7.6	6.57 ± 2.98			7.00 ± 3.28		

HADS-A, The Hospital Anxiety and Depression Scale-Anxiety. Significant of bold *p* values.

HADS-D, The Hospital Anxiety and Depression Scale-Depression.

### Source of COVID-19 knowledge

**Figure 2:**
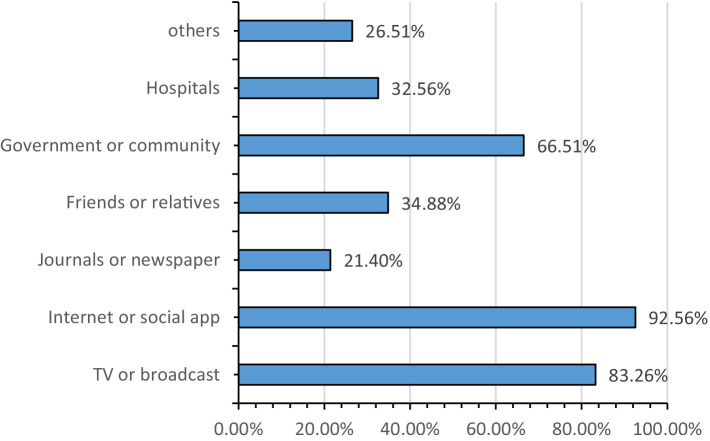
The sources of getting COVID-19 knowledge.

32.56% of IBD patients received information from hospitals. Internet/social apps and TV/broadcasts accounted for 92.56 and 83.26% of the sources of COVID-19 knowledge, respectively (details were shown in Figure 2).

### The knowledge of COVID-19 and its correlation with mental health

As reported in Table 3, most questions got over 60% of the correct rate. All participants knew which kind of mask to choose during coronavirus. 11.2% of participants knew which groups were more susceptible to COVID-19 infection. There was no significant difference in COVID-19 knowledge between patients with depression/anxiety scores ≥ 8 and scores < 8. Details were shown in Table 4.

**Table 3:** The knowledge of coronavirus in IBD patients.

Knowledge of coronavirus	Right rate	
	*N*	%
1. Which one is not an effective disinfectant for coronavirus?	71	33.0
2. Which one is the possible way of spreading Coronavirus?	129	60.0
3. The range of droplet propagation is?	133	61.9
4. The most important clinical manifestations of coronavirus are?	158	73.5
5. What is the medical observation period after encountering close contact with COVID-19 infection?	205	95.3
6. What groups of people are susceptible to COVID-19 infection?	24	11.2
7. Which one belongs to public measures to prevent coronavirus infection?	175	81.4
8. How should you deal with visible dirt on your hands?	4	1.9
9. In which situations should you wash your hands?	205	95.3
10. In which situation should you wear a mask?	205	95.3
11. What kind of mask should be selected during coronavirus?	215	100
12. Caution when using masks?	184	85.6
13. How often do masks need to be replaced?	156	72.6
14. How to check the air tightness of the mask?	177	82.3
15. How many times should windows be opened for ventilation per day?	68	31.6

IBD, Inflammatory Bowel Disease.

COVID-19, Corona Virus Disease 2019.

**Table 4:** The correlations between knowledge of COVID-19 and mental health.

IBD patients *N* (%)		Knowledge of COVID-19		
**Anxiety**				
Scores ≥ 8	57 (27)	49.91 ± 5.862	*Z* = −0.772	*p* = 0.440
Scores < 8	158 (73)	48.73 ± 9.255		
**Depression**				
Scores ≥ 8	73 (34)	47.33 ± 8.083	*Z* = −1.823	*p* = 0.068
Scores < 8	142 (66)	49.93 ± 8.588		

### Public self-prevention and correlation with mental health

Table 5 reported that during the COVID-19 pandemic, 72.1% of the IBD patients monitored their temperature at least once a day, 37.2% of the IBD patients went out at least once a week, and 53.8% of the IBD patients did not go to the hospital as much as possible per month. With increasing frequencies of monitoring temperature, hanging out, and going to the hospital, IBD patients reported higher scores for anxiety and depression, the effects were not significant (all *p* values > 0.05).

**Table 5:** The correlations between public self-prevention and mental health.

Questions		*n*	%	HADS-A		HADS-D	
Monitoring temperature (times/day)	0	60	27.9	5.35 ± 3.18	*H* = 8.983	5.87 ± 3.09	*H* = 2.590
	1–2	136	63.3	5.21 ± 2.82	**p = 0.011**	5.78 ± 3.54	*p* = 0.274
	3 and more	19	8.8	7.16 ± 2.97		7.00 ± 3.62	
Hanging out (times/week)	0	80	37.2	4.86 ± 3.11	*H* = 3.670	5.75 ± 3.86	*H* = 0.308
	1–2	87	40.5	5.68 ± 2.90	*p* = 0.160	5.94 ± 3.13	*p* = 0.857
	3 and more	48	22.3	5.88 ± 2.79		6.13 ± 3.24	
Going to hospital (times/month)	0	105	53.8	5.58 ± 2.78	*H* = 2.388	5.90 ± 3.59	*H* = 3.557
	1–2	80	41.1	4.88 ± 3.29	*p* = 0.303	5.75 ± 3.40	*p* = 0.169
	3 and more	10	5.1	5.40 ± 3.50		8.00 ± 2.11	

HADS-A, The Hospital Anxiety and Depression Scale-Anxiety. Significant of bold *p* values.

HADS-D, The Hospital Anxiety and Depression Scale-Depression.

### Multinomial logistic regression: demographic information, knowledge of COVID-19, public self-prevention, and life changes in anxiety or depression (scores ≥ 8)

The binary logistic regression reported that the omnibus tests of model coefficients was χ^2^ = 77.142, *p* values < 0.001, and *R*^2^ = 0.466. As shown in Table 6, IBD patients’ waiting time for admission, taking oral medicine regularly, and diet changes were positively correlated with anxiety or depression (all *p* values < 0.05). Timely periodic infusion of biological agents was negatively correlated with anxiety or depression (*p* value < 0.05).

**Table 6:** Multinomial logistic regression: demographic information, knowledge of COVID-19, public self-prevention, and life changes in anxiety or depression (scores ≥ 8).

Characters	*B*	OR	95% C.I.		*p* values
Living areas	0.321	1.378	0.954	1.990	0.087
Monitoring temperature (times/day)	0.580	1.787	0.934	3.419	0.080
Waiting time for admission (day)	1.305	3.688	1.003	13.554	**0.049**
Periodic infusion of biological agents	−0.535	0.586	0.413	0.830	**0.003**
Oral medicine administration regularly	2.913	18.407	1.975	171.530	**0.011**
Knowledge of COVID-19	−0.035	0.965	0.924	1.008	0.110
Diet changes	1.819	6.167	2.158	17.626	**0.001**
Constant	−2.973	0.051			0.072

COVID-19, Corona Virus Disease 2019. Significant of bold *p* values.

## Discussion

During the COVID-19 pandemic, fear of infection and the necessity of social distancing have affected people’s mental health (22). IBD patients are at high risk of infections and infectious complications because of malnutrition and immune-based therapies (23). We conducted a cross-sectional survey on the COVID-19 knowledge, public self-prevention measures, daily life changes, and mental health of IBD patients during the pandemic period. Consistent with previous study (23), poor mental health and a lack of management guidance for IBD under the impact of the COVID-19 pandemic were reported.

In detail, this study reported that Chinese IBD patients experienced a certain degree of anxiety and depression during the COVID-19 pandemic. This result was similar to that of Australian IBD patients (depression, 34.9%; anxiety, 32%) (10) and was consistent with the pooled incidence rate of anxiety (32.1%) and depression (25.2%) in worldwide IBD patients reported in 2021 (8). In addition, IBD patients in this study showed a higher incidence rate of anxiety and depression during the COVID-19 pandemic than before the COVID-19 pandemic (depression, 15%; anxiety, 20%) (24). The need for social distancing made face-to-face psychological counseling more difficult during the COVID-19 pandemic. And the limited access to medications and clinical specialist visits heightened IBD patients’ fears of infection (3). Thus, IBD patients’ mental health should be a focus.

IBD patients who lived in rural areas, with educational background lower than high school, and with a monthly income of less than 5,000 RMB showed a higher risk of getting depression or anxiety. However, these factors were not statistically significantly correlated with anxiety or depression. Rural areas had limited medical resources, a relatively weak ability of public health prevention measures, and a shortage of living materials (25). Lower income, high risks of contracting COVID-19, and social isolation were common risk factors for anxiety and depression (26). Most Chinese registered IBD centers are in cities, so it is hard for rural IBD patients to obtain medicine because of the longer distance. Besides, the chance of infection increases during long-distance travel to the hospital. Highly educated people had a better understanding of COVID-19 and were more active in obtaining information about COVID-19; therefore, they were more confident in facing the COVID-19 pandemic (27).

For IBD patients, dietary changes have been incorporated into therapeutic strategies (28). This study reported that a few IBD patients changed their diet, and diet changes showed as a risk factor for anxiety or depression. It implied that IBD patients seemed to have realized the importance of diet and strived to reduce their dietary changes. During the COVID-19 pandemic, general people got higher BMI and showed more snacks, sugary foods, and alcoholic beverages, less fruit and vegetable consumption (29). IBD patients showed less healthy diet changes (30, 31): fewer food choices and a higher risk of precipitous weight loss. Food supply decreased because of people hoarding food and the termination of production (32). This study showed that several participants increased or decreased the consumption of staple food, fresh vegetables, fruit, and meat/eggs/milk. Because of the limited number of participants, it is difficult to analyze which changes in diet are related to their anxiety or depression. More studies were needed to determine whether psychological factors affect dietary changes or whether dietary changes affect psychology. Anyway, maintaining food supply during public emergency events is crucial for IBD patients.

This study revealed that IBD patients decreased physical activity and increased sleep time during the COVID-19 pandemic. The changes in physical activity and sleeping time had no significant correlation with mental health (30, 33), and changes in physical activity did not influence the association between mental health and sleep (33). Differently, previous studies indicated that 40.5% of IBD patients reported worsened sleep during the COVID-19 pandemic (31), and sleep disturbances (such as difficulties with falling asleep, repeated wake-up, and early awakening) were statistically significantly associated with anxiety and depression (33). IBD patients who had anxiety and depression also were more likely to have worse daily functioning and lower physical activity (29, 31). Therefore, more studies are needed to focus on what kind of physical activity and sleep changes in IBD patients and how these changes affect their mental health.

This study indicated that more than 50% of IBD patients could take oral medicine regularly and receive timely periodic infusions of biological agents. Consistently, El-Dallal and colleagues found that less than 20% of IBD patients reported changing their biologic medication infusion schedules and medication regimens, respectively (31). Harris et al. (12) found that 87% of IBD patients did not change their IBD medication, and most of their services were largely uninterrupted. The situation was different in Italy: 94.5% of IBD patients had stopped or delayed biological treatment, and 5.5% had continued their therapy regularly (34). These differences may be related to the severity of the epidemic and quarantine policies in different countries. Moreover, this study showed that going to the hospital for periodic infusion of biological agents was a protective factor of depression or anxiety. Taking medicine regularly was not a significant factor for anxiety or depression in univariate analysis, but after controlling for other lifestyle factors, taking medication regularly was an independent risk factor for depression or anxiety. The reason may be that other factors in life change interacted with taking medication regularly. Unlike biologic agents which were infused on a monthly cycle, oral medicine was administrated daily. Daily medication needs caused higher pressure for IBD patients in the quarantine environments during the COVID-19 pandemic. According to Mohammad and colleagues’ study (35), patients showed higher adherence to health protocols than healthy people. Medicine and biological infusion are always emphasized in IBD therapies by doctors. Thus, ensuring the supply of routine treatment and medication for IBD patients would be good for alleviating their depression and anxiety.

This study showed that IBD patients had good motivation for knowing about COVID-19 and showed good implementation of public self-prevention measures. 53% of the IBD patients had received COVID-19-specific health education, and most of them thought that the provided knowledge was useful (10). However, most IBD patients got COVID-19 information from the internet/social app, TV, broadcast, government, and community, while not from the hospital in this study. IBD patients were still worried about the COVID-19 pandemic (56.0%), and the potential adverse effects of continuing immunosuppressants (36). 43.6% of IBD patients felt more vulnerable to COVID-19 infection due to their condition (43.6%) (37). This study reported that less than 30% of the IBD patients knew what kind of people are susceptible to COVID-19 and did not know how to deal with visible dirt on their hands, even though good hand hygiene is crucial to protect vulnerable patients (12). Although 90% of IBD patients received “at-risk” notifications from multiple sources, all IBD patients requested frequent updates of COVID-19 information from their gastroenterologists (12, 36). Therefore, the IBD patients lacked hospital support, especially for IBD self-management guidance in COVID-19.

Under the guidance of hospital gastroenterologists, general measures recommended for COVID-19 should focus on the specific needs of IBD patients. IBD patients preferred telemedicine to hospital visits and used telemedicine to address their concerns regarding COVID-19 infection and IBD (36). Establishing systemic online IBD self-management programs guided by hospitals has been encouraged, such as online guidance with hospital multidisciplinary cooperation and the supervision of patients’ skills through specific questionnaires (38). Telemedicine has been recommended during the COVID-19 pandemic and has been proven to reduce time to remission, outpatient visits, and hospital admissions (37, 39). It could also reduce the economic burden and infection risk for people living in remote rural areas and with poor economic. A previous study (40) indicated that the content of self-management programs should include medicine administration, proactively reducing transmission, managing hospital and healthcare facility exposure, infusion access, nutrition, reducing disease activity, smoking cessation, and vaccination. As the pandemic continues, the self-management of depression and anxiety should also be added to IBD self-management programs. Regardless, building a targeted self-management system for IBD patients during the COVID-19 pandemic is supported.

## Limitations

There were some limitations in this study. At first, this was a cross-sectional study, and the results cannot predict the development of mental health in IBD patients. Future prospective cohort studies are indispensable. Secondly, the reliance on self-reports may be prone to misinterpretation, and the remote electronic questionnaire made it hard for us to observe the filling process. Then, during the COVID-19 pandemic, IBD patients were often isolated at home, and whether their psychological status was closely related to the changes in their family support was not investigated in this study. At last, the participants in this study were from one hospital in central China, and the data on diet changes was small and limited the results of diet changes on mental health, so there is a need to expand the sample sources to different regions.

## Conclusion

To our knowledge, our study is the first cross-sectional study to assess the mental health outcomes, COVID-19 knowledge, public self-prevention measures, and life changes in Chinese IBD patients during the COVID-19 pandemic. We revealed that IBD patients knew about COVID-19 and showed good implementation of public self-prevention measures, but they lacked systemic targeted self-management and support from hospitals. We found that IBD patients’ diet changes, waiting time for admission, taking oral medicine regularly, and timely periodic infusions of biological agents were related to depression and anxiety.

## Data availability statement

The original contributions presented in the study are included in the article/supplementary material, further inquiries can be directed to the corresponding author.

## Ethics statement

The studies involving humans were approved by Third Xiangya Hospital of Central South University Ethics Board. The studies were conducted in accordance with the local legislation and institutional requirements. Written informed consent for participation in this study was provided by the participants’ legal guardians/next of kin.

## Author contributions

SH: Writing – review & editing, Writing – original draft. TX: Writing – original draft, Methodology, Investigation, Data curation. YX: Writing – original draft, Supervision, Software, Resources.
